# Hematoma, Hemarthrosis, and Hemotympanum: A Case Report of Acquired Hemophilia A

**DOI:** 10.5070/M5.52344

**Published:** 2026-07-31

**Authors:** Andrea Wolff, Ivy Dahlen, Arun Singavi

**Affiliations:** * Creighton University School of Medicine, Department of Emergency Medicine, Phoenix, AZ; ^ HonorHealth Cancer Care & Arizona Center for Cancer Care, Department of Medical Oncology, Chandler, AZ

## Abstract

A 75-year-old female with no prior history of bleeding disorders presented to the emergency department with spontaneous sublingual hematoma, hemarthrosis, hemotympanum, and cutaneous ecchymoses. She denied anticoagulant use, recent trauma, or underlying systemic disease. Initial laboratory findings revealed an isolated prolonged activated partial thromboplastin time (aPTT) that failed to correct with mixing studies. Factor VIII activity was markedly reduced, and the presence of factor VIII inhibitors was confirmed, leading to a diagnosis of acquired hemophilia A (AHA). Management included recombinant activated factor VII (rFVIIa) for hemostatic control, corticosteroids for immunosuppression, and weekly rituximab for inhibitor eradication. During her hospital course, serial monitoring demonstrated a decreasing inhibitor titer and improving coagulation profile. She was discharged and completed four weeks of rituximab and a steroid taper, with complete normalization of coagulation profile and inhibitor eradication within four weeks. This case underscores the importance of recognizing AHA in patients with unexplained bleeding and prolonged aPTT. Prompt diagnosis and targeted therapy are critical in reducing morbidity and mortality associated with this rare but potentially life-threatening disorder.^1^,^2^

**Topics:**

Acquired hemophilia A, spontaneous bleeding, factor VIII inhibitors, prolonged aPTT, Bethesda assay, recombinant activated factor VII (rFVIIa), immunosuppressive therapy.

**Figure:**
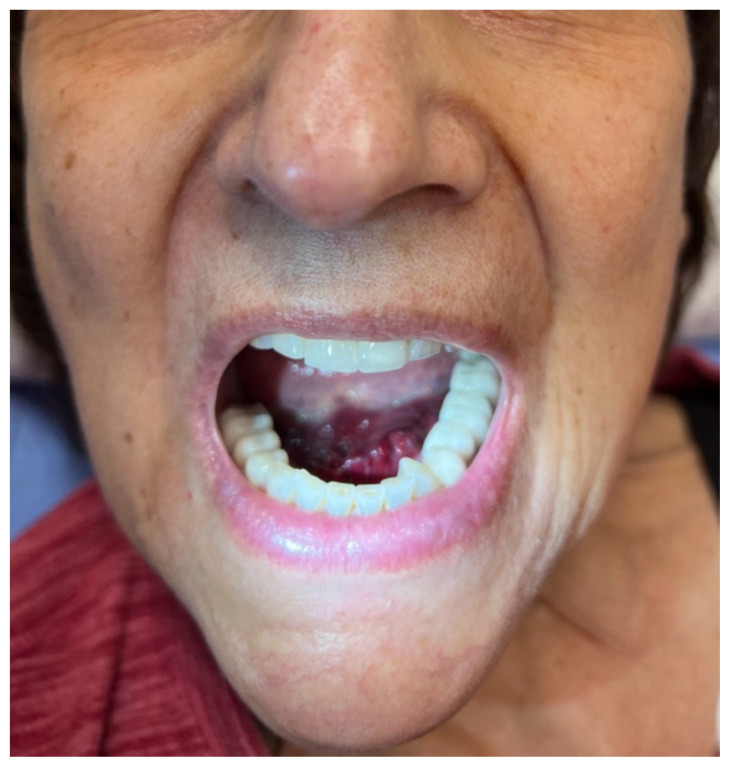


**Figure:**
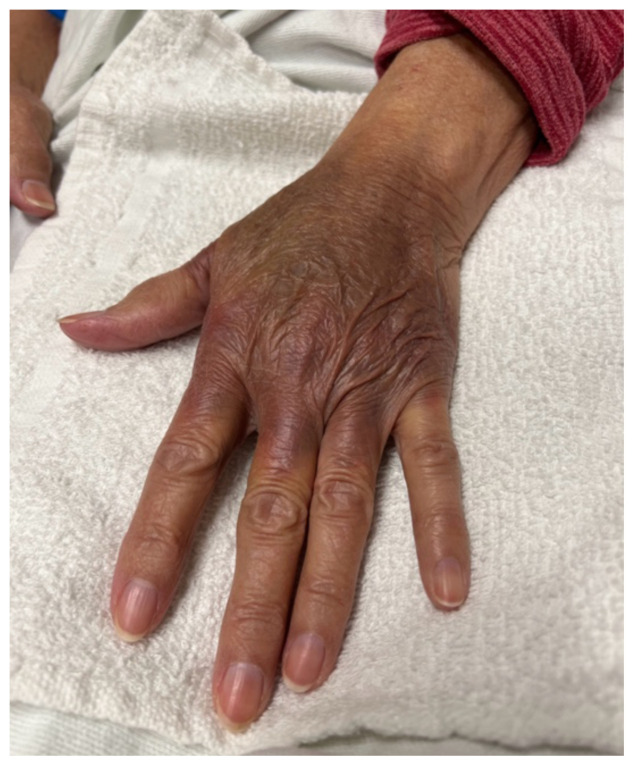


**Figure:**
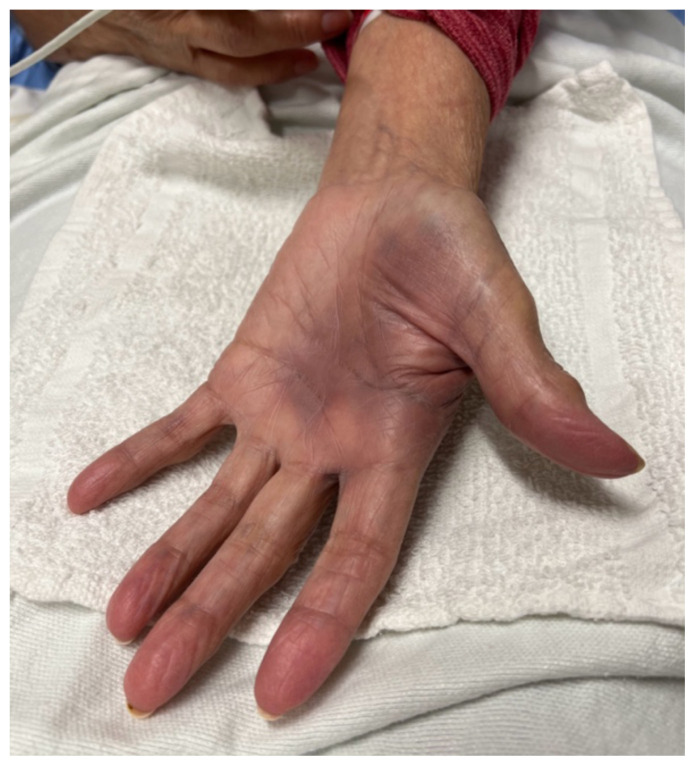


**Figure:**
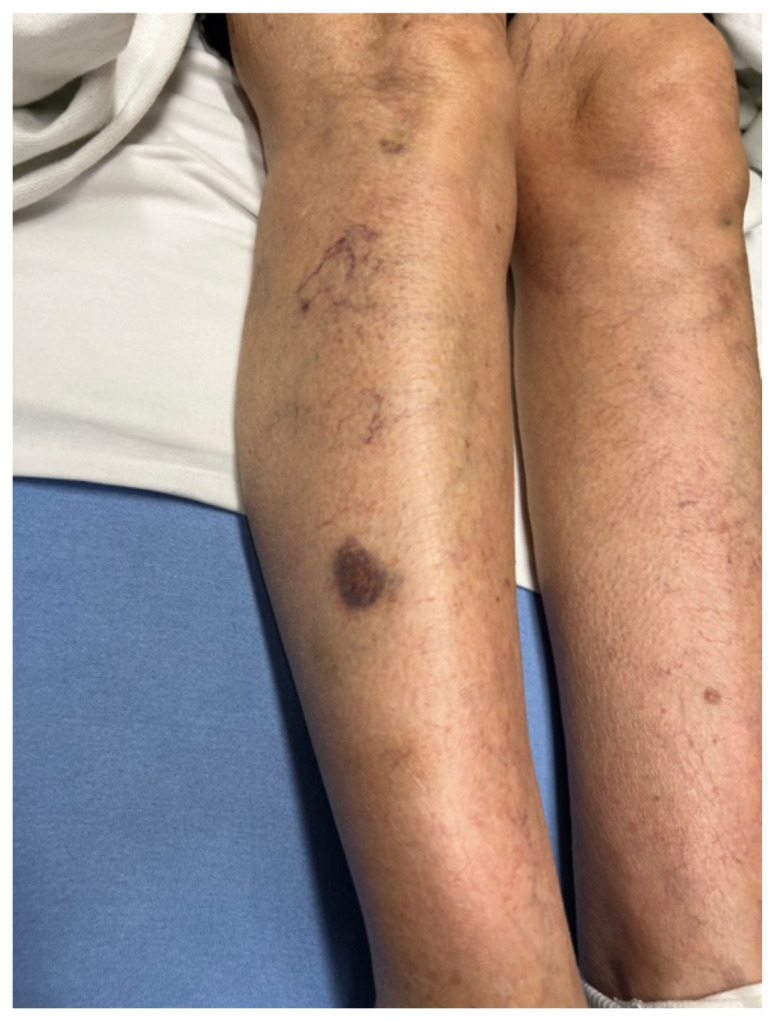


**Figure:**
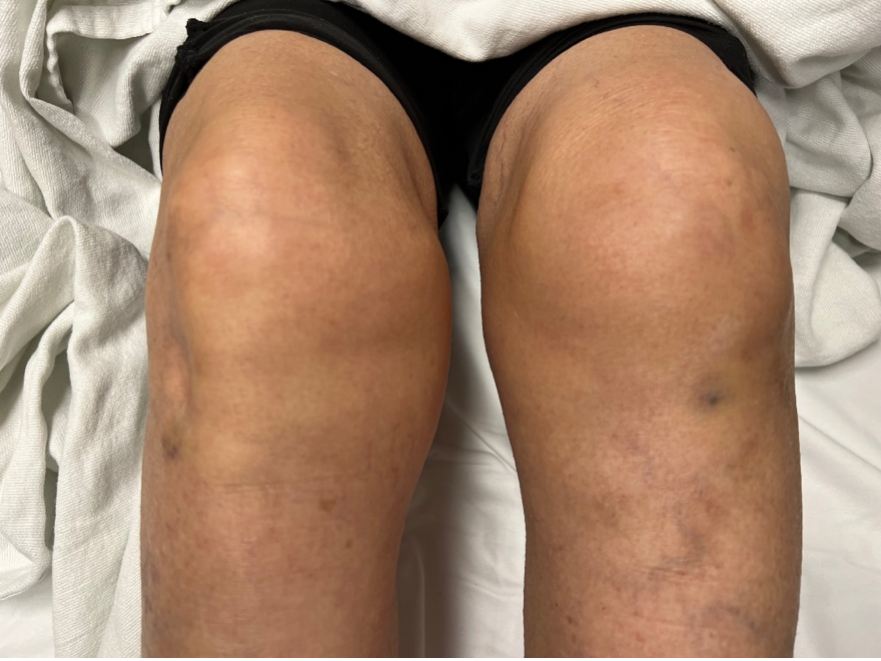


**Figure:**
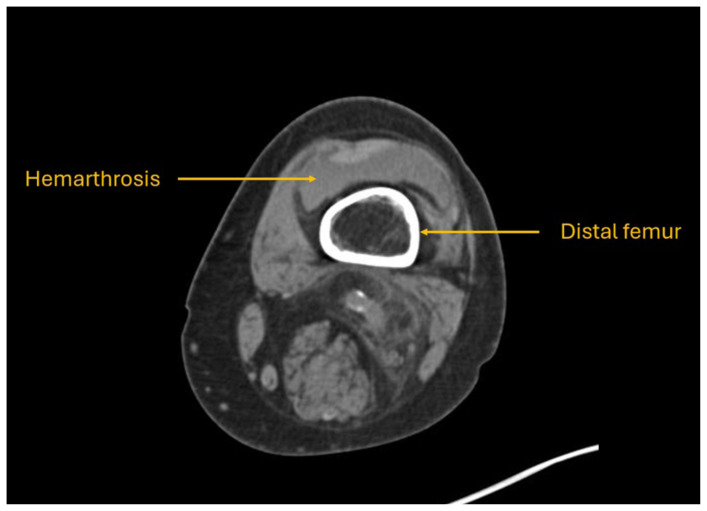


**Figure:**
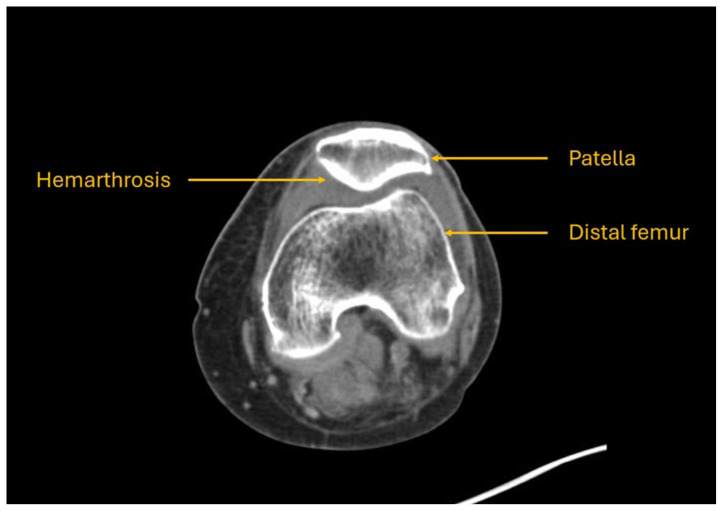
Video Link: https://youtu.be/YrKC6hq5Xuk

## Brief introduction

Acquired hemophilia A (AHA) is a rare autoimmune bleeding disorder characterized by the development of autoantibodies against factor VIII, leading to impaired coagulation and spontaneous hemorrhage.^3^ Unlike congenital hemophilia, which typically presents early in life, AHA occurs in individuals with no prior personal or family history of bleeding disorders and is most commonly diagnosed in the elderly.^1^,^4^ The estimated incidence of AHA is approximately 1.5 cases per million per year, and increases with age; AHA is most prevalent in adults over the age of 65, with a median age of 74–78 years.^1^,^3^,^5^ It can also be associated with malignancy, autoimmune disease, pregnancy, or infection, but up to 50% of cases are idiopathic with no clear inciting etiology.^4^ Due to its rarity and nonspecific clinical presentation, AHA is often misdiagnosed, leading to delays in appropriate treatment and increased morbidity and mortality.^1^

The pathophysiology of AHA involves the production of IgG autoantibodies that neutralize factor VIII, impairing coagulation and increasing the risk of spontaneous bleeding. While AHA may present with severe or life-threatening hemorrhage, bleeding often occurs in deep soft tissues, muscles, and mucous membranes.^1^,^4^ Unlike congenital hemophilia, AHA is not typically associated with hemarthroses.^1^ Laboratory findings characteristic of AHA include a prolonged aPTT that does not correct with mixing studies and significantly reduced factor VIII activity levels.^1^ The detection of factor VIII inhibitors via the Bethesda assay confirms the diagnosis.^4^

Management of AHA involves hemostatic control and immunosuppressive therapy. First-line hemostatic agents include bypassing agents such as recombinant activated factor VII (rFVIIa) or activated prothrombin complex concentrate (aPCC), which effectively control bleeding despite the presence of inhibitors.^5^ Once the diagnosis is confirmed and the deficient factor has been identified, administration of recombinant factor VIII may be considered.^8^ Immunosuppressive therapy (IST) aims to eradicate autoantibodies and typically consists of corticosteroids, cyclophosphamide, bortezomib, or rituximab.^7^,^9^ The median time to inhibitor eradication is approximately five weeks, but refractory cases may require additional immunomodulatory agents.^2^ Despite advancements in treatment, AHA remains associated with significant morbidity and a mortality rate of 15% to 40%, underscoring the importance of early diagnosis and prompt intervention.^4^

## Presenting concerns and clinical findings

A 75-year-old female presented to the emergency department complaining of bleeding underneath her tongue. She described a ten-day history of intermittent minor bleeding episodes, including spontaneous bruising of the right lower leg and the left hand. A few days prior to presentation, she noted some blood oozing from her right ear after using a cotton swab in the ear. She was seen at an urgent care and was treated with oral trimethoprim-sulfamethoxazole for presumed ear infection. The following day she described feeling a “pop” in the left knee while walking, and subsequently developed pain and swelling localized to the joint. On the day of presentation, she noticed development of a collection of blood under the tongue after brushing her teeth. She described mild difficulty and discomfort with swallowing but confirmed being able to swallow and to phonate without difficulty. She reported using ibuprofen for a few days for the discomfort and swelling of the knee but denied regular aspirin or anticoagulant use. She had recently started alendronate for osteopenia. She reported no traumatic injury, and denied any recent illnesses, other cardio-pulmonary symptoms, or symptoms of gastrointestinal bleeding. The patient did not report any prior history of malignancy, bleeding disorders, or coagulopathy. Her only reported prior medical diagnoses included osteopenia and an increase in unexplained falls over the last two to three years. Surgical history was significant for hysterectomy in the distant past. She was current on vaccinations and preventative cancer screenings, and denied any history of smoking, alcohol consumption, or illicit drug use.

On exam, the patient was awake, alert, and phonating normally with clear and articulate speech. She was managing her secretions without drooling or spitting and had no apparent respiratory compromise. Examination of the oropharynx demonstrated findings of a large sublingual hematoma without active extravasation (image 1). The floor of the mouth was soft, and no other intraoral lesions were identified. There was no trismus. Ocular examination was normal, but examination of the ears demonstrated scant dried blood within the right external auditory canal with mild hemotympanum, but no visible perforation of the tympanic membrane. Other significant findings included ecchymosis of the left hand (images 2 and 3), effusion of the left knee due to suspected spontaneous hemarthrosis (image 4), and numerous small ecchymoses over the lower legs (image 5).

Differential diagnoses included idiopathic thrombocytopenic purpura (ITP), thrombotic thrombocytopenic purpura (TTP), acquired von Willebrand’s disease, hematologic malignancy, vasculitis, medication-induced coagulopathy, and other acquired bleeding disorders.

## Significant findings

Complete blood count demonstrated a white blood cell count of 5.8 thousand/μL, hemoglobin of 12.2 g/dL, hematocrit of 36.7%, and the platelet count was 259 thousand/μL. Prothrombin time (PT) was within normal limits at 11.4 sec (INR 0.97), but Partial thromboplastin time (PTT) was elevated at 62.5 sec (normal range: 25.5 – 36).

Complete metabolic panel was within normal limits. Transaminases, bilirubin, and alkaline phosphatase levels were also within normal limits.

Subsequent hematologic evaluation was significant for a markedly abnormal factor VIII activity level of 3% (normal range 50 – 150%). Factor IX activity level was normal at 107% (normal 50 – 150%). Von Willebrand Factor Antigen and Von Willebrand factor activity levels were normal at 110% and 99%, respectively.

Examination of the oropharynx demonstrated a sublingual hematoma, which appeared as a slightly raised, dark red, somewhat nodular-appearing collection within the sublingual tissues without active bleeding, obscuring the sublingual frenulum and papillae. Ecchymosis of the left hand appeared as non-raised, reddish-purple discolorations overlying the dorsal and palmar surfaces of the hand with distal extension onto the proximal phalanges of the dorsal index, long, and ring fingers, and proximal extension to the dorsal wrist. Moderate swelling of the left knee relative to the right was noted; no erythema, deformity, or discoloration were present, but a punctate area of bruising was noted slightly proximal and lateral to the tibial tuberosity. The lower extremities demonstrated multiple small to moderately sized contusions, with the largest being localized to the right mid-lower leg anteriorly, measuring approximately 3–4 cm in diameter. Varicose veins were noted, without obvious thrombosis or active bleeding.

Non-contrast computed tomography (CT) scan of the left knee confirmed a small to moderate suprapatellar joint effusion with Hounsfield unit measurements suggestive of blood products (+47 Hounsfield units), consistent with acute hemarthrosis. The hemarthrosis appears as a gray, fluid-density collection slightly darker than the surrounding tissues that conforms to the suprapatellar space anterior to the femur, continuing distally between the femur and the patella.

Computed tomography of the soft tissue neck, chest, abdomen, and pelvis were obtained with contrast; these were unremarkable and demonstrated no evidence of acute bleeding nor suspicious masses to suggest malignancy. The patient has provided written consent for the publication of these images.

## Patient course

The patient was admitted to the hospital after immediate hematology consultation with a presumptive diagnosis of acquired hemophilia A. The patient was treated with intravenous dexamethasone and an initial trial of Fresh Frozen Plasma (FFP). Minimal change in her laboratory parameters resulted after FFP; therefore therapy with rFVIIa was initiated (initial dose 90 mcg/kg), with a reduction in PTT from 62.5 sec to 49.2 sec.

On hospital day #2, the patient was transitioned to oral corticosteroids (prednisone 1mg/kg) daily, and rFVIIa therapy was continued (70 mcg/kg every 3 hours for 5 doses) with improvement in aPTT to 39.9. Factor VIII activity level also improved to 11%. Flow cytometry and myeloma panel were negative. On hospital day #3, she reported improvement in her left knee pain and resolution of her dysphagia. Dosing of rFVIIa was decreased to 45 mcg/kg every 8 hours. Bethesda titer resulted at 4.3 Bethesda units, confirming presence of inhibitor. On hospital day #4, the aPTT stabilized at 44 seconds with no further bleeding sequelae. Therapy with rFVIIa was discontinued, and treatment with weekly rituximab was initiated.

The patient was discharged on day #6 with a five-week prednisone taper. She completed four weeks of rituximab, with complete normalization of laboratory parameters by week 4. On subsequent follow up at three months, the patient continued to do well with no recurrence of bleeding episodes.

## Discussion

Acquired hemophilia A is a rare and potentially life-threatening condition characterized by the development of autoantibodies against coagulation factor VIII, producing a deficiency in functional factor VIII activity and a predisposition to bleeding sequelae.^1^ However, as in the presented case, approximately 50% of cases are idiopathic, and a clear inciting factor cannot be identified.^2^,^3^,^6^

The clinical presentation of AHA varies widely. A minority of patients may be diagnosed while asymptomatic, with the condition being identified as an isolated elevated aPTT on routine laboratory diagnostics.^8^ In fact, a diagnosis of AHA may be the initial presentation of an underlying illness and can precede symptoms of the causative disease process.^8^ However, more commonly patients will present with bleeding sequelae, with severity ranging from minor cutaneous or mucosal bleeding to severe, potentially life-threatening hemorrhage. Minor bleeding associated with AHA typically involves subcutaneous and intramuscular hematomas and mucosal bleeding.^1^,^4^,^5^ Unlike congenital hemophilia, hemarthrosis is uncommon in AHA, but as in the case presented, these may occur in a minority of patients.^1^ Hemorrhage associated with AHA can be severe, most commonly gastrointestinal bleeding, retroperitoneal hematoma, and postpartum hemorrhage, and may require massive transfusion and aggressive efforts to achieve hemostasis.^2^,^4^ Compartment syndromes may occur when the limbs are affected.^8^ Severe hemorrhage may occur in up to 70–90% of patients, with a mortality rate of approximately 5–10% when such hemorrhage occurs.^2^

The overall mortality rate associated with AHA is high, estimated to be between 15 and 42%.^2^ This condition is frequently associated with underlying illnesses that themselves have a high mortality rate, such as malignancy and autoimmune disease, and primarily affects a vulnerable population of elderly adults more likely to have other comorbid disease. Treatment strategies that incorporate therapies such as immunosuppression or steroid use are also associated with potential complications such as an increased risk of infection, sepsis, and gastrointestinal bleeding.^2^,^4^ Lastly, the majority of patients have no prior history of coagulopathy; because AHA is often not recognized until laboratory data are available, the diagnosis and subsequent treatment are often delayed, further contributing to the high mortality rate associated with this disease.^2^ It is therefore critical to rapidly identify these patients early if they present with minor symptoms, prior to the development of acutely life-threatening hemorrhage.

The diagnosis of AHA should be suspected in patients presenting with new-onset, unexplained bruising or bleeding of any severity in the setting of a prolonged aPTT despite no previous history of hemophilia.^2^ Confirmation of the diagnosis requires laboratory evaluation (figure 1); initial evaluation will demonstrate normal platelet levels and a normal PT, with an isolated prolongation of the aPTT.^3^,^6^ This pattern is atypical of oral anticoagulant use or platelet disorders and should be recognized as a possible indication of AHA. Elevations of the aPTT can also occur in the presence of antiphospholipid antibodies (commonly called the “lupus anticoagulant” syndrome); however, this does not typically present with a bleeding diathesis.^3^,^6^ If addition of normal plasma causes the PTT to correct, a factor deficiency is present. Replacement of factors with FFP or supplementation with Vitamin K can help alleviate symptoms in these patients. However, if the addition of normal plasma fails to correct the PTT, an inhibitor is likely present.^3^ Measurement of factor VIII and factor IX activity levels can narrow the differential diagnosis to a specific factor, confirming the diagnosis.^3^ The Bethesda assay allows quantification of the concentration of factor VIII inhibitors (measured in Bethesda units [BU]); by mixing the patient’s plasma with normal plasma, the remaining factor VIII activity can be measured and is beneficial in guiding therapy.^1^,^11^

Initial management of patients with AHA will vary depending on clinical presentation and the severity of bleeding symptoms, but should prioritize control of acute blood losses.^1^,^2^,^8^ A substantial number of patients will present with severe or life-threatening hemorrhage as their initial presentation, at which time the diagnosis is often unknown.^2^,^4^,^8^ Aggressive hemostatic resuscitation during the initial evaluation and management should prioritize replacement of blood losses and control of hemorrhage.^1^,^2^,^8^ However, once the diagnosis is suspected, initiation of factor replacement with bypassing agents such as four-factor prothrombin complex concentrates (PCC) or recombinant activated factor VII (rAFVII) is crucial to achieving hemostasis.^1^,^3^,^8^,^13^ Repletion of factor VIII is impractical for initial management; the ability to measure factor VIII activity level is not available at many institutions, and is generally not available on an emergent basis, so definitive identification of the culprit factor is often delayed. Because these patients are at high risk of hemorrhagic complications, and there is substantial mortality associated with this diagnosis, patients with suspected or confirmed AHA typically require admission to the hospital with hematologic consultation.^1^

Once diagnosis is suspected and after initiation of bypass agents, corticosteroids should be started (prednisone 1 mg/kg/day) and tapered over several weeks.^2^ The goal of therapy is bleeding cessation and eradication of antibody (AHA remission).^14^ Most guidelines recommend the addition of adjunct therapies such as rituximab or cyclophosphamide, particularly in those with factor activity levels < 1% or high inhibitor levels (>20 BU).^14^ Rituximab is typically administered IV weekly over four weeks. There is emerging data on the use of emicizumab, a bispecific FVIII-mimetic antibody, instead of rituximab for more rapid hemostatic control and in patients with contraindications to immunosuppressive therapy or with refractory disease. 3 Emicizumab is administered subcutaneously weekly; however, availability in the inpatient setting may be limited.^15^

Monitoring parameters include aPTT, factor VIII activity levels and inhibitor assays. Patients can be considered for discharge once bleeding control is achieved without the use of bypass agents and factor VIII activity levels are rising.^1^,^3^,^8^,^14^

Outpatient follow up and completion of therapy is vital, along with continued monitoring.^4^ Eradication is demonstrated by normalization of Factor VIII activity levels and Bethesda assays.^16^ Relapses can occur even years after remission; therefore, patients need regular follow-up.^3^,^4^,^16^ Patients should have monthly factor VIII activity levels for six months, followed by quarterly for one year, and semi-annually thereafter for several years.^16^

This case of AHA underscores the importance of early recognition of rare but potentially devastating bleeding disorders in patients who present with unexplained bleeding symptoms. The timely and effective use of bypassing agents and immunosuppressive therapies, along with an understanding of the complex diagnostic and management strategies for AHA, is crucial in improving patient outcomes. The management of AHA can be challenging and requires a multimodal approach to control bleeding and address the underlying causative process. Initial management requires aggressive control of acute hemorrhage, often requiring the use of bypassing agents like rFVIIa or aPCC. However, the cornerstone of long-term treatment for AHA is immunosuppression aimed at eradicating the inhibitor and restoring factor VIII activity.^5^,^6^

## Supplementary Information




















